# IGFBP-4 tumor and serum levels are increased across all stages of epithelial ovarian cancer

**DOI:** 10.1186/1757-2215-5-3

**Published:** 2012-01-20

**Authors:** Rebecca A Mosig, Mollie Lobl, Emir Senturk, Hardik Shah, Samantha Cohen, Eugene Chudin, Robert Fruscio, Sergio Marchini, Maurizio D'Incalci, Ravi Sachidanandam, Peter Dottino, John A Martignetti

**Affiliations:** 1 Department of Genetics and Genomic Sciences, Mount Sinai School of Medicine, New York, NY, USA; 2 Division of Gynecologic Oncology, Mount Sinai School of Medicine, New York, NY, USA; 3 Prognosys Biosciences, La Jolla, California, USA; 4 San Gerardo Hospital, University of Milano-Bicocca, Monza, Italy; 5 Department of Oncology, Instituto "Mario Negri", Milano, Italy; 6 Mario Negri Gynecological Oncology Group (MaNGO), Milano, Italy

**Keywords:** IGFBP-4, epithelial ovarian cancer, serum biomarker, RNA-Seq, transcriptome

## Abstract

**Background:**

We sought to identify candidate serum biomarkers for the detection and surveillance of EOC. Based on RNA-Seq transcriptome analysis of patient-derived tumors, highly expressed secreted proteins were identified using a bioinformatic approach.

**Methods:**

RNA-Seq was used to quantify papillary serous ovarian cancer transcriptomes. Paired end sequencing of 22 flash frozen tumors was performed. Sequence alignments were processed with the program ELAND, expression levels with ERANGE and then bioinformatically screened for secreted protein signatures. Serum samples from women with benign and malignant pelvic masses and serial samples from women during chemotherapy regimens were measured for IGFBP-4 by ELISA. Student's t Test, ANOVA, and ROC curves were used for statistical analysis.

**Results:**

Insulin-like growth factor binding protein (IGFBP-4) was consistently present in the top 7.5% of all expressed genes in all tumor samples. We then screened serum samples to determine if increased tumor expression correlated with serum expression. In an initial discovery set of 21 samples, IGFBP-4 levels were found to be elevated in patients, including those with early stage disease and normal CA125 levels. In a larger and independent validation set (82 controls, 78 cases), IGFBP-4 levels were significantly increased (p < 5 × 10^-5^). IGFBP-4 levels were ~3× greater in women with malignant pelvic masses compared to women with benign masses. ROC sensitivity was 73% at 93% specificity (AUC 0.816). In women receiving chemotherapy, average IGFBP-4 levels were below the ROC-determined threshold and lower in NED patients compared to AWD patients.

**Conclusions:**

This study, the first to our knowledge to use RNA-Seq for biomarker discovery, identified IGFBP-4 as overexpressed in ovarian cancer patients. Beyond this, these studies identified two additional intriguing findings. First, IGFBP-4 can be elevated in early stage disease without elevated CA125. Second, IGFBP-4 levels are significantly elevated with malignant versus benign disease. These findings provide the rationale for future validation studies.

## Background

Epithelial Ovarian Cancer (EOC) is the most lethal female reproductive tract malignancy with nearly 200,000 new cases and > 125,000 deaths attributable to the disease each year worldwide [1]. The high fatality-to-case ratio is due, in part, to lack of effective screening modalities to detect ovarian cancer at an early stage wherein rates of cure exceed 90%. Most patients present with advanced stage disease and the cornerstone of treatment is surgical debulking followed by platinum-based chemotherapy. The other major contributor to the high fatality-to-case ratio is chemoresistant disease. In fact, while 80% of patients have a complete clinical response to their primary therapy, the majority will die from disease recurrence within 5 years. The overall worldwide 5-year survival rate of the disease is < 40% [2], however, when detected early, the 5-year survival rate more than doubles [3]. Unfortunately, EOC has non-specific, vague, gastrointestinal, and often ignored symptoms such as bloating, irregularity, and indigestion and there are no approved population screening methods, making early detection difficult and uncommon.

The search for reliable, specific, and sensitive serum-based biomarkers for EOC has a long history and its major highlight remains the identification of CA125 nearly 30 years ago [4]. Although CA125 is expressed in a majority (~80%) of late stage disease, it is elevated in only a subset (~50%) of early disease, thus limiting its usefulness for early disease detection [5]. In an attempt to overcome this limitation, proteomic-based studies have sought novel biomarkers. Examples of promising markers found through candidate and proteomic approaches include HE4, transthyretin, and CA72.4 [6-8]. Nonetheless, no markers are approved for population screening or disease detection whereas only CA125, along with HE4, are approved for monitoring of recurrent disease [9]. The history of poor performance of individual markers has led researchers to also evaluate panels or combination markers [6,7,10,11].

Next Generation Sequencing technologies, as applied to cancer genomes and transcriptomes, has allowed a relatively unbiased and more complete view of the global changes that define tumors [12,12-14]. Specifically, analyses of melanoma, pancreatic, lung, and breast cancers have revealed key pathways and genes affected in these cancers by mutations, copy number variations, and transcriptional changes [12,12-14]. Furthermore, application of this knowledge can be used to discover both personalized and global diagnostic and prognostic biomarkers [3,15]. We hypothesized that application of RNA-Seq technology to ovarian cancer could identify overexpression of secreted proteins that could act as novel biomarkers.

We analyzed the global gene expression patterns of a highly clinically annotated sample set of EOC representing both early and late stage tumors by RNA-Seq. Focusing specifically on transcripts that had evidence for secretion of their translated protein products, we identified IGFBP-4 to be highly expressed across all stages of EOC. IGFBP-4 is one of six IGFBP's, a family of regulators of normal and tumor cell biology [16], whose function is to inhibit IGF-I and -II binding to their receptors, IGF1R and IGF2R [17]. It is present in all body-fluids, secreted primarily by the liver, but also expressed by a number of organs, including the ovaries. In the ovary, it is involved in follicle selection and is upregulated *in vivo *and *in vitro *in response to estrogen [17]. Tumor expression of IGF family members has been linked to breast, endometrial, colon, and skin cancers [16]. In this study, we demonstrate that IGFBP-4 serum levels were significantly upregulated in primary and recurrent EOC patients even in a number of cases where CA125 levels were within normal limits.

## Methods

### Patients and Specimen Collection

EOC tumor samples were collected from Mount Sinai School of Medicine (New York, USA) and San Gerardo Hospital (Milan, Italy) patients at the time of surgery under their respective IRB-approved protocols. Samples were divided in the operating room and a portion sent for pathology confirmation and staging. Portions were flash frozen for RNA and protein analysis or used immediately for generating patient-derived cell lines. Papillary serous tumor samples were collected across all stages (five stage I/II, 11 stage III/IV, two disseminated peritoneal lesions, and two recurrent tumors). For comparison, two borderline tumors were also sequenced.

Blood samples were collected in gold top tubes (BD Biosciences, Franklin Lakes, New Jersey) directly prior to surgery, directly prior to chemotherapy, or at clinical office visits (controls), allowed to clot and centrifuged at 2600 rpm for 10 minutes to separate serum. Serum samples were aliquoted to minimize freeze thaw cycles and stored at -130°C. Patient characteristics including age, ethnicity, stage/grade of tumor are provided in Additional File 1: Table S1. Control samples were collected at the time of routine office visits.

For disease surveillance studies, blood samples were serially collected during each chemotherapy infusion, and in subsequent office visits thereafter. IGFBP-4 levels were measured for each visit, or averaged over the entire postsurgical period to give a composite value. Additional File 2: Table S2 highlights patient characteristics and treatment regimens. Disease recurrence status was assessed by a combination of positive CT/PET scans, CA125 levels, and/or positive operative laparoscopy.

### RNA extraction

RNA was extracted from frozen tissue using QIAzol according to the manufacturer's instructions (Qiagen, Valencia, California). Briefly, tissue was homogenized in QIAzol on ice. Chloroform was added, mixed and centrifuged to allow for separation and removal of the aqueous layer. RNA was precipitated in isopropanol overnight at -20°C. The suspension was centrifuged to pellet the RNA, washed with 75% ethanol and then resuspended in RNAase-free water. RNA integrity numbers (RINs) were determined (Agilent Bioanalyzer, Agilent Technologies, Santa Clara, California) and only RNA with a RIN score of ≥ 8.0 was submitted for next-generation sequencing.

### RNA-Seq

Epithelial ovarian cancer transcriptomes were prepared for paired-end sequencing using the Illumina GAII platform by the manufacturer's protocols and with a second size selection step to reduce ligation artifacts. Reads were aligned using the software program ELAND32 (provided with the Illumina sequencing platform). Expression levels were quantified by running ERANGE v. 3.0.2. [18]. For each gene, ERANGE reported the number of mapped reads per kilobase of exon per million mapped reads (RPKM).

### Quantitative Real-time Reverse Transcription PCR (qRT-PCR)

RNA-Seq data was confirmed by qRT-PCR. One microgram of RNA was reverse transcribed using the BioRad Iscript system (BioRad, Hercules, California). qRT-PCR was performed on an ABI PRISM 7900HT sequence detection system (Applied Biosystems, Carlsbad, California). Cycle number values were normalized against two housekeeping genes, B2M and GAPDH. Data shown are the averages of three separate experiments, each performed in triplicate. The IGFBP-4 primers used were IGFBP-4 Fwd: 5'- AGGTCCTTCCTTTAGGTCTG-3' and IGFBP-4 Rev: 5'- GGAAGACTTGAAGCACAGAG-3'.

### ELISA

Patient serum IGFBP-4 levels were analyzed in duplicate using Active IGFBP-4 ELISA (Diagnostic Systems Laboratories, Inc, a Beckman Coulter Company, Webster, Texas) according to the manufacturer's protocol. Standards and internal controls were assayed on each plate for calibration and consistency. Colorimetric absorbance was detected using a microplate reader at 450 nm with a background correction at 620 nm. A standard curve was generated for each plate and sample IGFBP-4 concentrations determined.

### Statistical Analysis

Statistical differences were determined using the Student's t test or ANOVA with Bonferroni correction and post-hoc test. ROC analysis was performed using SPSS software (IBM, Chicago, Illinois).

## Results

### IGFBP-4 is expressed in early, late, and recurrent EOC tumors

Paired-end sequencing reads were aligned to the genome and the number of reads mapping to a specific mRNA transcript expressed as coverage. Coverage adjusts the number of mapped reads by the overall length of the mRNA transcript as longer transcripts will necessarily have more mapped reads than a shorter transcript expressed at the same level. Approximately 10,000 transcripts were expressed at an average coverage level greater than one in the 22 tumor samples sequenced. This list was then cross-referenced against the Secreted Protein Database [19] to identify tumor-expressed genes that could be secreted into the serum and therefore useful as potential biomarkers. This analysis yielded a working dataset of ~1700 transcripts. It was necessary for the potential biomarker to be expressed by a majority of tumors and we therefore looked for candidates with minimal variation among samples. For this reason, transcripts with a standard deviation greater than 75% of the average expression level were disqualified from further analysis (68%, 1169 transcripts). The remaining 541 transcripts (32%) were designated "top candidate genes". And from this list, we selected IGFBP-4 as a "proof-of-principle" candidate based on pathway analysis, proposed upregulation in other cancers as well as the availability of a commercial antibody. Importantly, we found no previous reports examining serum IGFBP-4 in ovarian cancer in the literature.

IGFBP-4 ranked among the top 7.5% (average top 3%) of expressed genes across all tumor samples. The average coverage levels between the different tumor types are shown in Table 1. For a comparison and validation of our system, we examined the expression level of a number of previously described ovarian cancer markers. CA125 averaged in the top 8% (average coverage level 18.426). Interestingly, HE4 was highly expressed; it was on average in the top 10 genes expressed with an average coverage level of 435.8. Putative markers CA72.4 and transthyretin were not present in our top 10% gene list.

**Table 1:** RNA-Seq tumor coverage values

Disease Group	Mean (StDev)	Range	N#
Borderline	55.035 (14.425)	44.835-65.235	2
Early EOC	18.796 (16.450)*♮	4.280-37.895	5
Late EOC	18.745 (14.412)*♮	6.335-60.005	11
Disseminated	13.893 (10.737)*♮	6.300-21.485	2
Recurrent	54.923 (17.999)	42.195-67.650	2

Coverage units is transcripts, adjusted to the full length of the mRNA transcript of IGFBP-4.

Statistical significance was tested using a Student T-test. *statistically different from borderline;

♮ statistically different from recurrent tumors

From the same tumor RNA samples that were sequenced, cDNA was synthesized and RT-PCR used to validate expression profiles (Additional File 3: Table S3). To confirm our RNA-Seq findings, we analyzed an independent validation set of an additional 22 tumor samples by qRT- PCR. IGFBP-4 was again highly expressed in all samples (data not shown).

### Serum IGFBP-4 is elevated in EOC patients even those with normal CA-125

**Figure 1:**
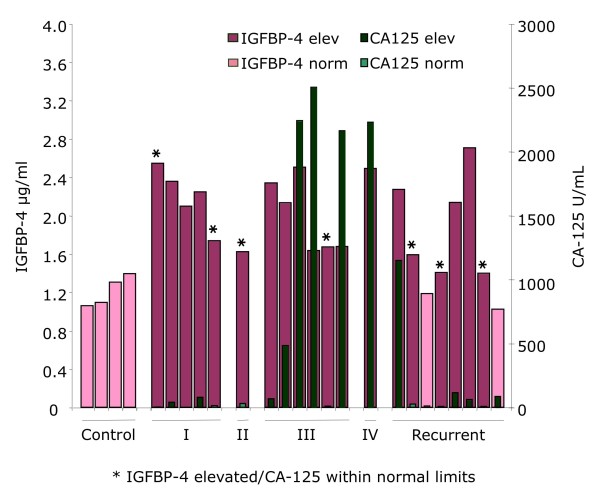
**IGFBP-4 is upregulated in patient serum samples compared to controls**. Patient and control serum samples were analyzed by ELISA and IGFBP-4 concentrations determined and graphed (pink bars). Samples with IGFBP-4 levels less than control values are light pink and IGFBP-4 levels greater than controls are dark pink. As a comparison, CA125 levels for patients are graphed in green. Using the standard cutoff of 35 U/ml, CA125 levels were divided into high (dark green) and low (light green). Those patients where IGFBP-4 was a better marker (low CA125 but high IGFBP-4) are indicated with an asterisk.

To quantify IGFBP-4 serum protein expression levels in patients compared to controls, ELISA assays were used. We first chose a subset of "discovery" samples to analyze IGFBP-4 serum levels. In this initial analysis, we found the 21 patient samples (five stage I, one stage II, six stage III, one stage IV, and eight recurrent EOC) to have significantly higher levels of IGFBP-4 compared to controls (Figure 1, p < 0.0001). All of the primary samples and all but 2 of the 8 recurrent EOC samples had levels greater than that of the controls. Although the sample size for these data was limited, they provide the rationale for future studies including larger numbers of patients.

Given that CA125 is historically non-informative in ~20% of women with ovarian cancer at the time of their initial diagnosis, we also sought to compare the sensitivity of IGFBP-4 to that of CA125 [20]. Of interest, while eight of the 21 cases had CA125 levels within normal limits, seven of these had elevated IGFBP-4 levels (Figure 1, cases highlighted with an asterisk). Conversely, of the two recurrent patients with low IGFBP-4, only one had elevated CA125 while the other had a CA125 below threshold (Figure 1).

**Figure 2:**
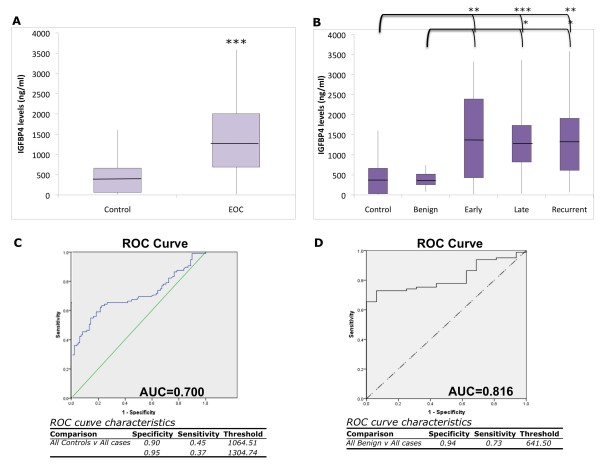
**Serum IGFBP-4 level analysis**. (A, B) Box and whisker graphs shows average (central line), quartile levels (top and bottom of box), minimum and maximum (ends of whiskers) IGFBP-4 levels in each group. (A) All controls (healthy and benign) compared to all stages of EOC (***p < 5 × 10^-7^). (B) Early, late and recurrent EOC groups have significantly higher IGFBP-4 levels than control or benign groups (*p < 0.05, **p < 0.005). See Table 2 for average values and ranges. Serum IGFBP-4 level ROC analysis of (C) all controls (healthy and benign samples) and all EOC cases (early, late, and recurrent) results in an AUC of 0.700. (D) ROC curve comparing benign controls only to all cases resulted in an AUC of 0.816. Corresponding sensitivity, specificity, and threshold values are listed below each curve.

Based on these results, we then analyzed a larger validation cohort consisting of 82 healthy controls and 78 cases. Cases consisted of 6 patients with benign ovarian disease, 16 early EOC (stage I/II) cases, 40 late EOC (stage III/IV) cases, and 16 recurrent cases (Additional File 1: Table S1). EOC cases again had significantly higher levels of IGFBP-4 than healthy and benign controls (*p < 0.05, **p < 5 × 10^-7^, ***p < 5 × 10^-11 ^Figure 2A, B). EOC (all stages) had an average IGFBP-4 of 1344.09 ng/ml compared to 400.9 ng/ml for healthy controls and 394.6 ng/ml for benign controls (Table 2).

**Table 2:** Serum IGFBP-4 levels

Disease Group	Mean (StDev)	Range	N#
Control	400.95 (470.8)	13.5-1602.8	82
Benign	394.60 (226.8)	83.2-744.9	6
Early EOC	1334.50 (1117.7)*♮	20.9-3323.3	16
Late EOC	1305.40 (889.3)*♮	41.4-3360.1	40
Recurrent	1450.4 (1039.3)*♮	65.3-3583.2	16

Units are μg/ml. Statistical significance was tested using a Student T-test. *Statistically different from control; *♮ *statistically different from benign

To analyze the sensitivity and specificity of IGFBP-4 as a marker for EOC we next performed ROC (Receiver Operating Characteristics) analysis. An ROC curve defining controls as both healthy and benign patients and cases as all EOC (early, late, and recurrent) had an area under the curve (AUC) of 0.700 (Figure 2C, p < 0.0005). Setting the specificity at 90%, sensitivity was 45.3%, with a threshold value of 1064.5 ng/ml. When the specificity was increased to 95%, sensitivity became 36.8% and the threshold was set at 1304.7 ng/ml.

Given the interest and clinical relevancy in differentiating between benign and malignant adnexal masses prior to surgery, we also compared benign versus cancer. When we used the benign cases alone as the control group, sensitivity increased at the high specificities required for high positive predictive values (Figure 2D). The ROC curve had an AUC of 0.816 and the threshold value for 94% specificity was 641.5 ng/ml yielding a sensitivity of 73%.

### IGFBP-4 as a disease surveillance biomarker

To analyze the potential of IGFBP-4 to monitor for disease recurrence and chemotherapeutic response, we collected serum samples from 10 patients undergoing chemotherapy following their surgical tumor removal (one stage I, one stage II, five stage III, two stage IV, and one recurrent). Patient characteristics are provided in Additional File 2: Table S2. Serum levels were tested during chemotherapeutic regimens in order to monitor the correlation between tumor status and IGFBP-4 levels. Our hypothesis was that IGFBP-4 levels may provide insight into tumor behaviour, ie resistance or recurrence. Follow-up after final serum collection was between 7 and 16 months. Patients were triaged into two groups, no evidence of disease (NED, n = 5) or alive with disease (AWD, n = 5). Follow-up period did not vary significantly between groups (Table 3, 13.2 months in AWD, 11.8 months in NED, p = 0.592) although the NED group was significantly younger (Table 3, NED average age 48 years, AWD average age 58.8 years, p < 0.005) and represented earlier stage disease than the AWD group (Table 3, average stage 3.2 in AWD, 2.6 in NED, p = 0.305)

**Table 3:** Follow-up patient characteristics

Group	Age (Range)*	Stage	Follow-up
AWD	58.8 (55-65)	3.2	13.2
NED	48 (44-55)	2.6	11.8

^a^Average stage found by assigning values to each stage as follows: Stage I = 1, II = 2, III = 3, IV = 4, Recurrent = 5; *p < 0.005

**Figure 3:**
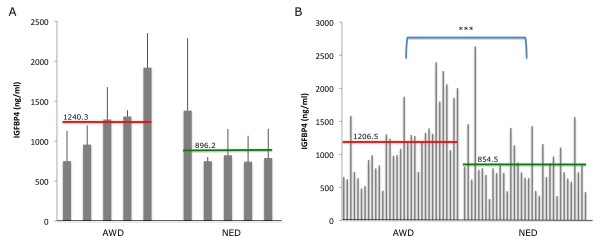
**Serum IGFBP-4 levels increased in EOC post-operative patients with recurrent or persistent disease**. A) Cumulative average IGFBP-4 serum levels for patients assessed as either NED or AWD. Average value for each group is shown as horizontal green (NED) or red (AWD) lines, p = 0.178. B) Serial serum IGFBP-4 measurements for NED or AWD patients. Average value for each group is shown as horizontal green (NED) or red (AWD) lines, ***p < 0.005.

The average IGFBP-4 levels of each patient following surgery trended lower in the NED compared to the AWD group (p < 0.10). Most interestingly, 58% (19/33) of the IGFBP-4 AWD measurements were above the 1000 ng/ml threshold (determined in ROC analysis above) compared to only 23% (8/35) in the NED group. The average IGFBP-4 reading for the NED group was significantly less than the AWD group (NED = 854.49, AWD = 1206.53 ng/ml, p < 0.005, Figure 3).

## Discussion

Using whole transcriptome analysis across all stages of EOC, we initially identified IGFBP-4 as a secreted protein highly expressed in all tumors. We then confirmed and quantitated IGFBP-4 overexpression in patient serum samples. To our knowledge, this is the first report demonstrating increased serum IGFPB-4 expression in ovarian cancer patients.

Despite large and significant differences between mean IGFBP-4 levels in cases and controls, ROC analysis revealed limited sensitivity at the specificities required for a simple single marker ovarian cancer screening test (Figure 2C, D). This is due to the level of overlap between the controls and cases which makes differentiating the two groups more complex. Above all, the low occurrence rate of EOC, combined with invasive nature of first-line treatment (surgical cytoreduction combined with chemotherapy), require a very high specificity, suggested to be at least 99.6% and a sensitivity of at least 75% to yield a positive predictive value of 10 [21]. Nonetheless, our studies suggest that, with further study, determination of IGFBP-4 levels could provide use in three clinical settings.

First, as shown in Figure 1, IGFBP-4 serum levels can be significantly increased in cases of early- and late-stage disease even when CA125 is within normal limits. Notably, three of the six early-stage cases with normal CA125 levels had increased IGFBP-4 levels. Therefore, combining IGFBP-4 and CA125 could increase the sensitivity for detecting EOC, especially, early stage disease. Future studies will be required.

Second, it has now been recommended that women with a suspicious adnexal mass should be referred to a gynecologic oncologist for evaluation since the early distinction between a benign and malignant mass represents an important clinical decision point. [reviewed in [22]].. In our patient cohort, malignant masses were associated with average IGFBP-4 levels ~3× higher than benign masses (Figure 2). When assay specificity is set at 94%-the highest we could achieve, given the number of samples in our dataset -, sensitivity is 73%. Increasing the sample sizes for these studies will increase the power of the analysis and will allow us to better analyze the overlap between and variability within groups. It will therefore be of future interest to increase sample sizes and re-evaluate the clinical utility of IGFBP-4 alone or in combination with other markers for distinguishing between benign and malignant masses.

Finally, we investigated the potential use of IGFBP-4 as a biomarker to monitor disease recurrence and resistance to treatment in patients receiving chemotherapy. While levels did not reach statistical significance, there was a trend for NED patients to have lower average IGFPB4 levels compared to AWD patients. Although levels in chemotherapy patients did not always remain below the ROC-determined threshold of 1000 ng/ml, those whose cumulative IGFBP-4 level average was less than the threshold were more likely to be in the NED group than those with higher averages (Figure 3). Moreover, the percentage of serial IGFBP-4 readings above threshold was higher in the AWD group compared to the NED group, again suggesting that high serum levels of IGFBP-4 may be indicative of disease state. Although differences in age and stages between the two groups (Additional File 2: Table S2) may contribute to this difference, we believe it unlikely given that in our larger diagnostic data set there was no difference between early EOC IGFBP-4 levels and later stage disease levels (early average 1334.5 ng/ml, late average 1305.4 ng/ml, p = 0.91, Figure 2). Additionally, we did not find any correlation between age and IGFBP-4 levels in either cases or controls (Additional File 4: Figure S1, R^2 ^= 0.003), although a positive correlation has been previously reported in healthy individuals [23]. Finally, it should be noted that the chemotherapy regimens received by women with disease recurrence were not always uniform between the groups. In such a small sample set, and in such a novel study, it is unknown at this time what effect, if any, these differences in agents may have had on IGFBP-4 levels.

Future studies are planned primarily to increase sample size and diversity of patients. Unexpectedly, we noted that Hispanic cases have significantly higher serum IGFPB-4 levels compared to other non-Hispanic cases or all controls (Additional File 5: Table S4). We are unaware of previous reports or studies suggesting a biologic basis for this finding. Thus, this intriguing finding will be specifically explored in future studies.

The IGF pathway has been implicated in carcinogenesis [16] and the role of IGFBP-4 has been studied in a number of human malignancies, including lung, endocrine (thyroid and adrenal), breast, prostate, and hepatocellular cancers [17]. Increased serum IGFBP-4 levels have also been associated with breast cancer, melanoma, and acute lymphoblastic leukemia [17,24]. While no previous reports have examined IGFBP-4 serum levels in ovarian cancer patients, IGFBP-4 was one of 52 proteins identified in a proteomic analysis of EOC ascites fluid although serum levels were neither evaluated nor compared with control samples [25]. At this time it is unclear how increased levels of IGFBP-4 may relate to initiation or progression of ovarian cancer. Our findings may initially seem counterintuitive, as the understood role of IGFBP-4 is to bind to and inhibit IGF-I and IGF-II, thereby suppressing cell growth and proliferation. In cancer settings, however, it is suggested to do exactly the opposite [17,24]. One possible explanation pertains to the hormone responsiveness of IGFBP-4. In an estrogen-rich environment that may occur as a result of ovarian cancer, IGFBP-4 is thereby over stimulated, and could serve as a marker for this subset of cancers. In a second scenario, involving the known role of IGFBP-4 in follicle stimulation, IGFBP-4 becomes constitutively expressed over years of repeated ovulation, and this continued expression might drive overgrowth of the epithelial cell layer of the ovary, contributing directly to the growth of the tumor. At best, these hypotheses are speculative, and biologic proof is required. However, given the suggestion of diagnostic and prognostic significance, as well as the potential as a therapeutic or preventative target, we believe these future studies worthwhile. These studies may work specifically to address issues of samples size, adjust for patient ethnicity and investigate the molecular role of IGFBP-4 is carcinogenesis of progression.

## Conclusion

In sum, our studies have identified that serum IGFBP-4 is on average upregulated ~3-fold in EOC cases compared to either healthy population controls or in those women with benign ovarian masses. This upregulation is present across all stages of EOC, including early stage disease and in women with recurrence of their cancer following treatment. Finally, IGFBP-4 levels can be elevated in women with early stage disease, whose CA125 levels are within normal limits. Based on these findings, we believe IGFBP-4 represents an interesting candidate biomarker for detection and surveillance of papillary serous ovarian cancer.

## List of abbreviations

EOC: Epithelial Ovarian Cancer; IGFBP-4: Insulin-like growth factor binding protein; NED: No Evidence of Disease; AWD: Alive With Disease.

## Competing interests

Eugene Chudin and Mark Chee are employees and shareholders of Prognosys Biosciences, Inc. All other authors declare no conflicts of interest.

## Authors' contributions

RAM participated in sample collection, selection, sequencing analysis, and all molecular studies and manuscript drafting. ML participated in all molecular studies. ES participated in sample collection and sequencing analysis. HS provided bioinformatics support and analysis. SC supplied samples and clinical information. EC provided bioinformatics support and analysis. RF, SM, and MD supplied samples and clinical information and participated in study design. RS provided bioinformatics support and analysis as well as data interpretation. PD supplied samples and clinical information and participated in study design. JAM participated in overall study design, sample selection, sequencing analysis, and manuscript drafting. All Authors reviewed and approved the final version of the manuscript.

## Supplementary Material

Additional file 1 **Table S1**. Supplementary Table 1: Patient and tumor demographics. Click here for file

Additional file 2 **Table S2**. Supplementary Table 2: Patient demographic and chemotherapeutic data. Click here for file

Additional file 3 **Table S3**. Supplementary Table 3: qRT-PCR tumor coverage values. Click here for file

Additional file 4 **Figure S1**. Serum IGFBP-4 levels are not significantly correlated with age. Scatter plot of serum IGFBP-4 levels against age of both cases and controls shows no correlation between to the two. Click here for file

Additional file 5 **Table S4**. Supplementary Table 4: IGFBP-4 and age by ethnicity. Click here for file
