# Supplementary material for: IGFBP-4 tumor and serum levels are increased across all stages of epithelial ovarian cancer
Source: J Ovarian Res. 2012 Jan 20;5:3. doi: 10.1186/1757-2215-5-3 (PMC3271973; doi:10.1186/1757-2215-5-3)
Supplement: Additional file 1 — Table S1. Supplementary Table 1: Patient and tumor demographics. [file 1757-2215-5-3-S1.PDF]

Additional File 1: Table S1

Supplementary Table 1: Patient and tumor demographics

Stage	number	Ethnicity (%)						Age (Range)
		Ashkenazi	Asian	Black	Hispanic	White	Unknown	
**Control**	**82**	**7 (9%)**	**2 (2.5%)**	**2 (2.5%)**		**45 (55%)**	**26 (31%)**	**52.4* (25-83)**
**Benign**	**6**	**2 (33%)**	**1 (17.5%)**			**2 (33%)**	**1 (17.5%)**	**49.8* (21-74)**
**All Early**	**16**	**3 (19%)**		**1 (6%)**		**9 (56%)**	**3 (19%)**	**55.5 (38-82)**
early CCC	2	1				1		
early Endo	7	1				5	1	
early Muc	1			1				
early Pap	5	2				3		
**All Late**	**40**	**11 (27.5%)**		**4 (10%)**	**7 (17.5%)**	**14 (35%)**	**4 (10%)**	**61.8 (41-89)**
late non-pap	3	1			2			
late pap	33	10		4	5	11	7	
**All Recurrent**	**16**	**8 (50%)**	**1 (6%)**	**1 (6%)**	**2 (13%)**	**4 (25%)**		**56.1* (44-69)**

**Significantly less than late stage patients*
