# Supplementary material for: IGFBP-4 tumor and serum levels are increased across all stages of epithelial ovarian cancer
Source: J Ovarian Res. 2012 Jan 20;5:3. doi: 10.1186/1757-2215-5-3 (PMC3271973; doi:10.1186/1757-2215-5-3)
Supplement: Additional file 2 — Table S2. Supplementary Table 2: Patient demographic and chemotherapeutic data. [file 1757-2215-5-3-S2.PDF]

Additional File 2: Table S2

Supplementary Table 2: Patient demographic and chemotherapeutic data

Patient	Status	Age	Stage	Ethnicity	Chemotherapy
PT113	AWD	55	III	Ashkenazi	Taxol-Carboplatin
PT087	AWD	61	III	Caucasian	Taxol-Carboplatin
PT168	AWD	55	III	Caucasian	Avastin
PT034	AWD	65	IV	Ashkenazi	Gemzar-CPT11
PT079	AWD	58	III	Ashkenazi	Gemzar-Avastin-Oxaliplatin
PT047	NED	44	IV	Caucasian	Taxol-Carboplatin-Avastin
PT128	NED	48	I	Caucasian	Taxol-Carboplatin
PT066	NED	45	II	Caucasian	Taxol-Carboplatin
PT065	NED	48	III	Ashkenazi	Cytoxan-Avastin
PT105	NED	55	III	Ashkenazi	Taxol-Carboplatin-Avastin
