# Supplementary material for: IGFBP-4 tumor and serum levels are increased across all stages of epithelial ovarian cancer
Source: J Ovarian Res. 2012 Jan 20;5:3. doi: 10.1186/1757-2215-5-3 (PMC3271973; doi:10.1186/1757-2215-5-3)
Supplement: Additional file 3 — Table S3. Supplementary Table 3: qRT-PCR tumor coverage values. [file 1757-2215-5-3-S3.PDF]

Additional File 3: Table S3

Supplementary Table 3: qRT-PCR tumor coverage values

Disease Group	Mean (StDev)	Range	N#
Borderline	0.213 (0.030)	-	1
Early EOC	0.190 (0.089)	0.102-0.306	5
Late EOC	0.572 (0.862)*♮	0.024-2.528	11
Disseminated	0.795 (0.684)*♮	0.134-1.500	3
Recurrent	0.150 (0.084)	0.091-0.209	2
