# Supplementary figures and images for: IGFBP-4 tumor and serum levels are increased across all stages of epithelial ovarian cancer
Source: J Ovarian Res. 2012 Jan 20;5:3. doi: 10.1186/1757-2215-5-3 (PMC3271973; doi:10.1186/1757-2215-5-3)

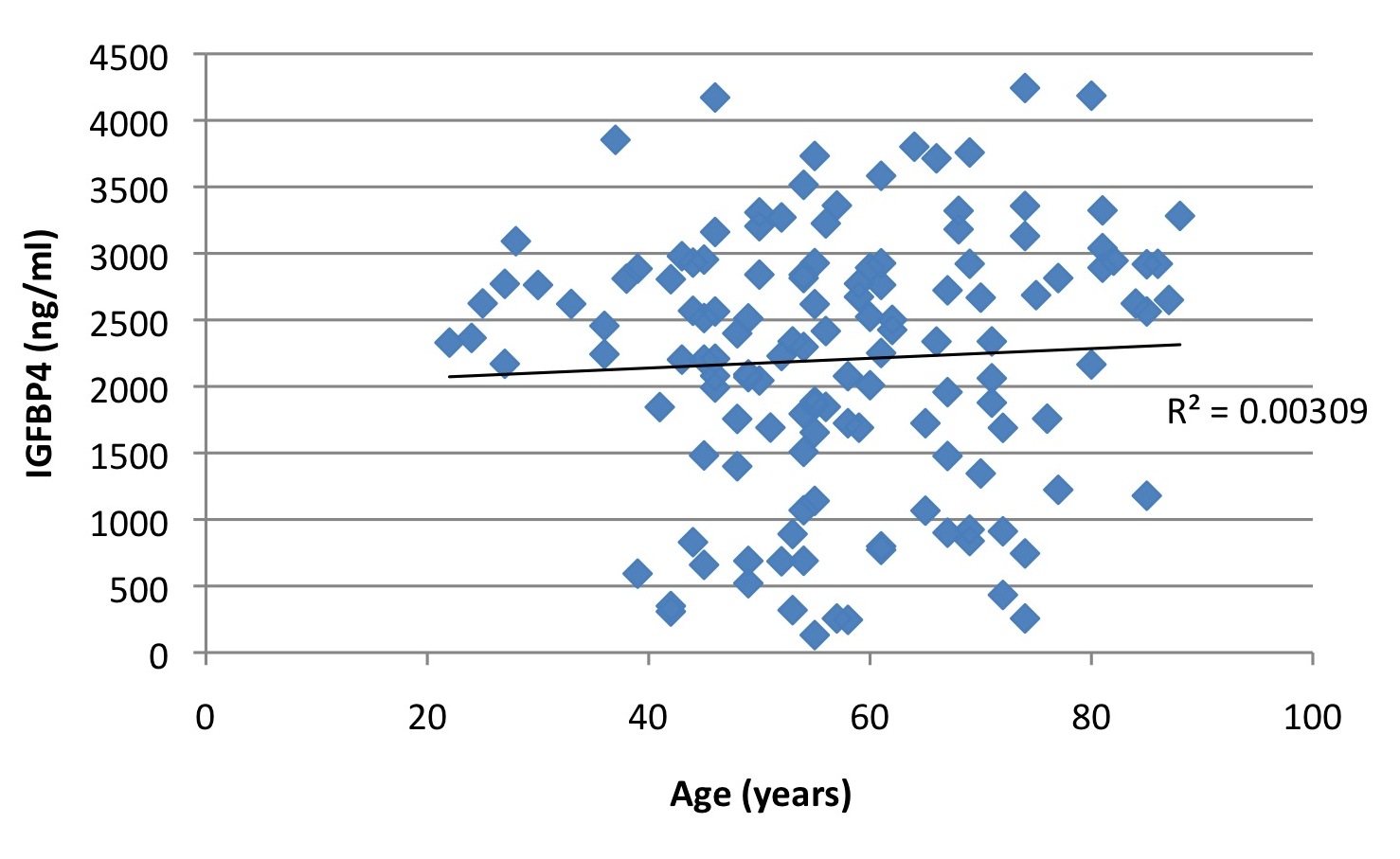

Supplement: Additional file 4 — Figure S1. Serum IGFBP-4 levels are not significantly correlated with age. Scatter plot of serum IGFBP-4 levels against age of both cases and controls shows no correlation between to the two. [file 1757-2215-5-3-S4.JPEG]
