# Supplementary material for: IGFBP-4 tumor and serum levels are increased across all stages of epithelial ovarian cancer
Source: J Ovarian Res. 2012 Jan 20;5:3. doi: 10.1186/1757-2215-5-3 (PMC3271973; doi:10.1186/1757-2215-5-3)
Supplement: Additional file 5 — Table S4. Supplementary Table 4: IGFBP-4 and age by ethnicity. [file 1757-2215-5-3-S5.PDF]

Additional File 5: Table S4

Supplementary Table 4: IGFBP-4 and age by ethnicity

Ethnicity	Cases			Controls		
	n	IGFBP4	Age	n	IGFBP4	Age
Ashkenazi	27	1049.43	54.56*	9	538.94	43.56**
Asian	1	1755.89	48.00	3	252.66	49.33
Black	7	727.20*	51.869*	2	816.45	50.00
Hispanic	10	1721.20	63.10			
White	29	1332.57	60.76	48	441.65	58.69
Unknown	14	1 (6%)830.70*	58.80	32	242.05	50.04**

*statistically different from Hispanic; **statistically different from Whites
